# Association Between the Backhand Technique and the Prevalence of Dominant-Arm Elbow Pain and Low Back Pain in Tennis Players: A Cross-Sectional Study

**DOI:** 10.7759/cureus.106135

**Published:** 2026-03-30

**Authors:** Saverio Colonna, Giuseppe Postorino, Matteo Tognon

**Affiliations:** 1 Rehabilitation Medicine, Spine Center, Bologna, ITA; 2 Research and Development, Spine Center, Bologna, ITA

**Keywords:** backhand technique, dominant-arm elbow pain, low back pain, one-handed backhand, overuse injuries, sports biomechanics, tennis elbow, tennis injuries, tennis players, two-handed backhand

## Abstract

Background

Tennis is a physically demanding sport characterized by explosive movements, repeated trunk rotations, and asymmetric loading of the dominant upper limb. Overuse injuries are common, with dominant-arm elbow pain (DEP) and low back pain (LBP) representing frequent musculoskeletal complaints among tennis players. Biomechanical differences between the one-handed backhand (1HB) and the two-handed backhand (2HB) may influence the distribution of mechanical loads on the upper limb and spine; however, their relationship with clinically relevant symptoms remains unclear. This cross-sectional observational study aimed to investigate the association between the backhand technique and the prevalence of DEP and LBP in a heterogeneous population of tennis players.

Methodology

An anonymous online questionnaire was distributed through tennis clubs and social media between February and November 2025. A total of 455 responses were collected, of which 445 complete questionnaires were included in the final analysis.

Results

The mean age of the participants was 35.1 ± 17.9 years, and 73.9% were male. The median weekly training volume was five hours (interquartile range = 3-11). In crude analyses, elbow pain was more frequent among players using the 1HB (56.5% vs. 32.9%, p < 0.001), while LBP was slightly more common in the same group (59.5% vs 49.5%, p = 0.049). However, multivariable Poisson regression models adjusted for age, sex, training volume, competitive level, and years of practice showed no independent association between the backhand technique and DEP (prevalence ratio (PR) = 1.08; 95% confidence interval (CI) = 0.80-1.46; p = 0.625) or LBP (PR = 1.18; 95% CI = 0.95-1.48; p = 0.142). Stratified analyses revealed that the 1HB was significantly more common among male players (p < 0.001), and elbow pain was also more prevalent in males (p = 0.00076), whereas the prevalence of LBP did not differ significantly between sexes (p = 0.798). Among participants reporting elbow symptoms (n = 190), 64.7% localized pain to the lateral epicondylar region and 35.3% to the medial epitrochlear region. Overall, the backhand technique was not independently associated with either elbow or lumbar symptoms.

Conclusions

These findings support a multifactorial model of injury risk in tennis, suggesting that factors such as age, sex, cumulative load, and player characteristics may play a greater role than the isolated choice of the backhand technique.

## Introduction

Tennis is a sport characterized by high physical demands, involving explosive movements, repeated trunk rotations, and asymmetric loading of the dominant upper limb. Overuse injuries represent the primary musculoskeletal problem in tennis, with an overall prevalence reaching up to 47% among athletes across both competitive and recreational levels [1].

Among the most frequently reported chronic conditions are upper-extremity tendinopathies. The most commonly described disorders include dominant-arm elbow pain (DEP), rotator cuff injuries, and shoulder impingement, with epicondylitis recognized as one of the most typical overuse injuries affecting the dominant arm of tennis players [2].

The literature provides limited evidence describing the epidemiology of injuries in both junior and senior tennis players [3-8]. As a result, the overall understanding of injury patterns in tennis remains incomplete, which may inadvertently influence both the management and clinical care of players.

In 2011, both the Association of Tennis Professionals (ATP) and Women’s Tennis Association (WTA) tours implemented a web-based medical documentation system designed to record injuries. Data from this ATP online medical documentation system reported relatively stable injury rates between 2014 and 2017, ranging from 1% to 3% annually [1]. Injury distribution by anatomical region during surveillance between 2012 and 2016 indicated that the most frequently affected musculoskeletal areas included the spine, shoulder, foot/ankle complex, and elbow [1].

DEP, often associated with lateral epicondylopathy (tennis elbow) and/or medial epicondylopathy (golfer’s elbow), represents an overuse condition affecting the tendons of the forearm. This disorder may develop in a substantial proportion of tennis players as a consequence of repeated muscular contractions occurring during stroke execution [9].

Several studies [10] have described lateral epicondylopathy as a generally self-limiting condition, with approximately 89% of diagnosed patients reporting a reduction in pain after one year [11]. However, other investigations have estimated that up to 40% of affected individuals experience prolonged symptoms [12] that may result in persistent functional impairment.

In the general adult population (30-64 years old), the prevalence of lateral epicondylitis is estimated at approximately 1.3% [13]. However, in recreational tennis players, the lifetime prevalence of DEP may approach 50%, suggesting a potential contribution of sport-specific loading and stroke mechanics [14,15].

Low back pain (LBP) is a common musculoskeletal condition among athletes and is frequently associated with compressive and torsional loads on the lumbar spine generated by repetitive movements such as the serve and rotational strokes. Among tennis players, discipline-specific epidemiological data show a certain degree of heterogeneity. In a cross-sectional analysis comparing adult tennis players with non-playing controls, Saraux et al. [16] did not find a significantly higher risk of LBP in tennis players than in controls, suggesting that participation in tennis alone does not necessarily lead to a higher prevalence of LBP compared with the general population.

In contrast, other studies [5,6,17,18] indicate that LBP represents a clinically relevant problem in tennis players, although the available evidence varies depending on age group and study methodology. In a prospective study [17] involving 198 competitive adolescent tennis players (SMASH cohort), 43% of athletes reported at least one episode of LBP during 52 weeks of monitoring. The incidence of a first episode was 0.91 per 1,000 hours of tennis training or match play (95% confidence interval (CI) = 0.85-0.97), while the overall incidence, including recurrences, was 2.11 events per 1,000 hours of exposure.

In another prospective study conducted in tennis players, LBP was identified as one of the most common and recurrent complaints [5], although notable differences were observed in the underlying etiology between adolescent and adult athletes. In an earlier study involving 148 professional tennis players, Marks et al. reported that 38% of athletes had missed a tournament due to LBP, while 29% reported chronic episodes of LBP [18]. These findings provide relevant estimates of LBP prevalence in young athletes, where the duration of sports participation and technical experience may influence the development of lumbar symptoms.

Background

Some authors have suggested that the repeated execution of the serve, particularly due to trunk hyperextension during the acceleration phase, may contribute to the development of injuries affecting the lumbar spine [19]. However, modern tennis requires repeated accelerations and decelerations combined with substantial trunk rotation, making the biomechanics of both the forehand and backhand strokes critical determinants of performance as well as injury risk.

The backhand, in particular, represents a key technical component that can be performed either with a one-handed backhand (1HB) or a two-handed backhand (2HB), each characterized by distinct kinematic and kinetic patterns. Genevois et al. [20] demonstrated that clear differences exist in the motor patterns of the 1HB and 2HB, particularly regarding the contribution of the non-dominant arm and the segmental coordination of the upper limb. The 1HB relies on a longer lever arm and requires greater control and stabilization of the dominant limb, potentially increasing mechanical stress on the upper extremity [21].

Several biomechanical studies [21,22-24] have shown that the 2HB involves a different pattern of energy generation and transfer along the kinetic chain, with greater contributions from trunk and pelvic rotation compared with the 1HB, potentially leading to increased torsional loading on the lumbar and thoracic spine during stroke execution. This greater rotational loading may predispose players to symptoms of LBP due to increased stress on myofascial tissues, intervertebral joints, and the deep stabilizing structures of the spine [25-27].

At the same time, the backhand stroke has been associated with common musculotendinous overload conditions such as DEP, particularly in the presence of repeated eccentric loading patterns affecting the wrist and forearm extensors of the dominant limb [28-32]. The findings of an epidemiological study [7] also suggest a potential association between the backhand technique and the occurrence of DEP. However, while DEP has been investigated in relation to technical and equipment-related variables, the literature examining the interaction between backhand type, rotational loading of the spine, and the combined risk of LBP remains limited.

These biomechanical differences have led to the hypothesis that the backhand technique may influence the prevalence of specific overuse disorders, particularly DEP and LBP. However, although the kinematic characteristics of the 1HB and 2HB have been widely described, their relationship with clinically relevant outcomes has been only partially explored in heterogeneous populations of tennis players. Therefore, the present study aimed to investigate the association between the backhand technique (1HB vs. 2HB) and the prevalence of DEP and LBP in a broad population of recreational and competitive tennis players.

We hypothesized that the 1HB, by reducing trunk rotational contribution while increasing load on the dominant upper limb, might be associated with a lower prevalence of LBP but a higher prevalence of elbow symptoms. In addition, as a secondary outcome, the study evaluated the prevalence of elbow pain location within the sample, distinguishing between lateral symptoms (tennis elbow) and medial symptoms (golfer’s elbow), and their relationship with the use of 1HB or 2HB.

## Materials and methods

This cross-sectional survey was conducted to investigate the association between the backhand technique and the prevalence of DEP and LBP in tennis players. Participants were asked to report symptoms specifically associated with playing tennis rather than general musculoskeletal pain. No specific criteria regarding pain intensity or duration were provided; responses were based on participants’ subjective interpretation.

The study was conducted by disseminating the initiative through social media and by contacting tennis club organizers via email and direct communication, inviting them to encourage their members to participate in the survey between February 2025 and November 2025. Participation consisted of completing an anonymous questionnaire on a voluntary basis. The full questionnaire administered through Google Forms is provided in the Appendices.

Eligible participants were adult tennis players who practiced tennis at recreational or competitive levels and voluntarily completed the online questionnaire. Participation was anonymous, and no identifiable personal data were collected.

To facilitate and expedite completion, the questionnaire was implemented using Google Forms and distributed as a digital survey. Participants were able to complete the questionnaire directly from their smartphone or computer through an anonymous online platform. Responses were automatically collected and organized into a spreadsheet database (Microsoft Excel) for subsequent analysis.

A total of 455 questionnaires were received. Ten questionnaires with incomplete key variables were excluded from the final analysis, resulting in a final sample of 445 participants. Implausible values were screened and excluded from the analysis.

Competitive level was self-reported and categorized into the following five hierarchical groups: beginner/recreational player, amateur competitive player (Tennis Progetto Rating Amatoriale (TPRA)/Unione Italiana Sport Pertutti (UISP) circuits), third category, second category, and professional (ATP/WTA circuit). Due to the recruitment strategy (social media and club-based dissemination), the response rate could not be determined.

The study was conducted in accordance with Good Clinical Practice (GCP) standards and the principles of the Declaration of Helsinki. As no identifiable personal data were collected and participation was fully anonymous, formal Institutional Review Board (IRB) approval was not required according to applicable regulations.

Statistical analysis

The collected data were organized into contingency tables showing the number of individuals in each category. For each outcome (DEP and LBP), a 2 × 2 contingency table was constructed, with one dimension representing the backhand type and the other representing the presence or absence of the condition.

The chi-square test was used to evaluate whether a statistically significant association existed between the backhand type and the presence of DEP or LBP. This test compares the observed frequencies with those expected under the assumption that the variables are independent. Statistical significance was defined as a p-value <0.05.

The Cochran-Armitage trend test was used to evaluate the linear association between competitive level and the proportion of players using the 1HB. Subgroup analyses were performed to explore potential differences across competitive levels and pain location; these analyses were exploratory, not pre-specified, and not adjusted for multiple comparisons.

To examine the association between the backhand technique and musculoskeletal symptoms while controlling for potential confounding factors, multivariable Poisson regression models with robust variance were used to estimate prevalence ratios (PRs) for DEP and LBP. Poisson regression with robust variance was used to directly estimate PRs, as the outcomes of interest were common, and logistic regression may overestimate associations when outcome prevalence is high. The models included the following independent variables: backhand technique (1HB vs. 2HB), age and years of tennis practice (both entered as continuous variables), sex (male/female), weekly training volume (hours per week), and competitive level.

## Results

The baseline characteristics of the overall study population are reported in Table 1.

**Table 1:** Baseline characteristics of the study population (n = 445). 1HB = one-handed backhand; 2HB = two-handed backhand; SD = standard deviation; IQR = interquartile range

Variable	Value
Total participants	445
Age, years, mean ± SD	35.1 ± 17.9
Weekly training volume, hours, median (IQR)	5 (3–11)
Years of practice, median (IQR)	14 (7–24)
Male sex, n (%)	329 (73.9%)
Female sex, n (%)	116 (26.1%)
1HB, n (%)	168 (37.8%)
2HB, n (%)	277 (62.2%)

When stratified by the backhand technique (Table 2), significant differences emerged between players using a 1HB and those using a 2HB.

**Table 2:** Baseline characteristics stratified by the backhand type. Values are presented as mean ± standard deviation (SD), median (interquartile range), or number (%). Differences between groups were assessed using Student’s t-test for continuous variables with normal distribution, the Mann–Whitney U test for non-normally distributed variables, and the chi-square (χ²) test for categorical variables. 1HB = one-handed backhand; 2HB = two-handed backhand; DEP = dominant-arm elbow pain; LBP = low back pain

Variable	1HB (n = 168)	2HB (n = 277)	Test statistic	P-value
Age, years, mean ± SD	48.8 ± 14.9	26.8 ± 14.0	t = 15.7	<0.001
Male sex, n (%)	151 (89.9%)	178 (64.3%)	χ² = 34.1	<0.001
Weekly training volume, hours, median (IQR)	4 (3–6)	6 (3–12)	Z = −2.45	0.014
DEP, n (%)	95 (56.5%)	91 (32.9%)	χ² = 24.7	<0.001
LBP, n (%)	100 (59.5%)	137 (49.5%)	χ² = 3.87	0.049

Players in the 1HB group were significantly older and more frequently male compared with 2HB players. Weekly training volume also differed between groups, with 2HB players reporting a higher median weekly training volume. In crude analyses, elbow pain was more prevalent among 1HB players, and LBP was also slightly more frequent in the 1HB group (Table 2).

Given the marked difference in the use of the 1HB between male and female players, and the independent association between male sex and elbow pain in the multivariable analysis, a stratified descriptive analysis of technique and symptom prevalence by sex was performed (Table 3).

**Table 3:** Distribution of the backhand technique and musculoskeletal symptoms by sex. The table reports the proportion of players using the 1HB, the prevalence of DEP, and the prevalence of LBP stratified by sex. P-values were calculated using the chi-square test for comparison between male and female players. Percentages are calculated within each sex. 1HB = one-handed backhand; 2HB = two-handed backhand; DEP = dominant-arm elbow pain; LBP = low back pain

Sex	n	1HB (%)	DEP (%)	LBP (%)
Male	329	46.3	46.6	52.2
Female	116	14.2	28.3	54.2
P-value (male vs. female)	—	<0.001	0.00076	0.798

The use of the 1HB was significantly more frequent among male players than female players (p < 0.001). DEP was also more prevalent in males (p = 0.00076), whereas the prevalence of LBP did not differ significantly between sexes (p = 0.798) (Table 3). These marked baseline differences, particularly in age and sex distribution, suggested the presence of potential confounding factors and justified the use of multivariable regression models to estimate adjusted PRs.

In multivariable Poisson regression models with robust variance for the overall cohort, adjusted for age, sex, weekly training volume, competitive level, and years of practice, backhand type (1HB vs. 2HB) was not significantly associated with either DEP or LBP (Table 4). For LBP, one amateur category (TPRA/UISP) showed a significantly lower prevalence (p < 0.01) (Table 4).

**Table 4:** Multivariable Poisson regression models with robust variance (adjusted PRs). The model was adjusted for age, sex, weekly training volume, competitive level, and years of practice (n = 445). Results should be interpreted as associations in a cross-sectional design. 1HB = one-handed backhand; 2HB = two-handed backhand; DEP = dominant-arm elbow pain; LBP = low back pain; PR = prevalence ratio; CI = confidence interval; TPRA = Tennis Progetto Rating Amatoriale; UISP = Unione Italiana Sport Pertutti

DEP			
Variable	Adjusted PR	95% CI	P-value
1HB vs. 2HB	1.08	0.80–1.46	0.625
Age (per year)	1.02	1.01–1.03	<0.001
Male sex	1.57	1.04–2.37	0.032
Weekly training volume (per hour)	0.99	0.97–1.00	0.056
Third category vs. reference	0.67	0.46–0.98	0.040
Amateur/Beginner vs. reference	0.48	0.30–0.78	0.003
LBP			
Variable	Adjusted PR	95% CI	P-value
1HB vs. 2HB	1.18	0.95–1.48	0.142
Age (per year)	1.01	0.99–1.02	0.214
Male sex	1.12	0.89–1.40	0.321
Weekly training volume (per hour)	1.00	0.98–1.02	0.874
TPRA/UISP vs. reference	0.63	0.47–0.85	0.003

For DEP, independent associations were observed for older age and male sex, both of which were associated with a higher prevalence of elbow pain. Conversely, some competitive categories showed a lower prevalence compared with the reference category (Table 4).

Among participants reporting DEP (n = 190), 123 (64.7%) localized their symptoms to the lateral epicondylar region, whereas 67 (35.3%) reported medial epitrochlear pain. In exploratory analyses stratified by pain location, backhand type was not independently associated with either lateral or medial elbow pain (Table 5).

**Table 5:** Location of elbow pain and association with the backhand technique. Exploratory Poisson regression models with robust variance adjusted for age, sex, weekly training volume, and competitive level. Analyses were restricted to participants reporting elbow pain who specified pain location (n = 190). 1HB = one-handed backhand; 2HB = two-handed backhand; PR = prevalence ratio; CI = confidence interval

Outcome	Adjusted PR (1HB vs. 2HB)	95% CI	P-value
Lateral elbow pain (epicondylar region)	1.15	0.82-1.61	0.41
Medial elbow pain (epitrochlear region)	0.98	0.66-1.45	0.92

In exploratory analyses stratified by pain location, backhand type was not independently associated with either lateral or medial elbow pain after adjustment for age, sex, weekly training volume, and competitive level. These findings indicate that the absence of association between the backhand technique and elbow symptoms remained consistent regardless of pain location.

Trend in one-handed backhand use across competitive levels

When competitive levels were ordered hierarchically, and ATP/WTA (professional) players were included as the highest category, the proportion of players using the 1HB showed a decreasing trend as the competitive level increased. The Cochran-Armitage trend test confirmed a significant linear association (Z = −3.863; p = 0.0001).

**Figure 1:**
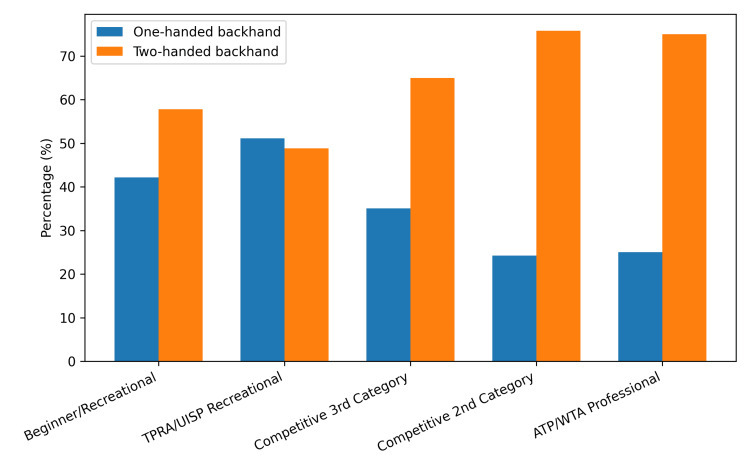
Distribution of the backhand technique across competitive levels. The proportion of the one-handed and two-handed backhands across increasing competitive levels, ordered from the lowest to the highest category. TPRA = Tennis Progetto Rating Amatoriale; UISP = Unione Italiana Sport Pertutti; ATP = Association of Tennis Professionals; WTA = Women’s Tennis Association

The prevalence of 1HB use (95% CI) by category was as follows: beginner/recreational, 42.2% (30.9-54.4; N = 64); TPRA/UISP, 51.1% (42.7-59.5; N = 131); third category, 35.0% (27.6-43.3; N = 137); second category, 24.2% (16.7-33.7; N = 95); and ATP/WTA (professional) 25.0% (12.7-43.4; N = 28) (Figure 1).

Overall, the likelihood of using the 1HB decreased as the competitive level increased. The wider CI observed in the ATP/WTA group reflects the smaller sample size but does not alter the direction of the overall trend.

Age and competitive level

The mean age differed significantly across competitive levels (Kruskal-Wallis test, p < 0.05). A significant negative correlation was observed between age and ordered competitive level (Spearman’s rho < 0, p < 0.05), indicating that players competing at higher levels (e.g., second category, first category, or ATP/WTA circuits) tended to be younger than those in amateur or TPRA/UISP groups. This pattern confirms the presence of a progressive age gradient across competitive categories.

Analysis by competitive category

Stratified analysis by competitive level revealed that the prevalence of the investigated conditions was not uniform across categories (overall chi-square test p < 0.05). This finding suggests that competitive level, and therefore likely factors such as age and training load, may influence the distribution of these conditions.

Dominant-arm elbow pain

At lower competitive levels (NC, amateur, TPRA/UISP), a higher prevalence of elbow complaints was observed among players using the 1HB, although these differences did not reach statistical significance (p > 0.05). In higher competitive categories (second and first categories), this difference tended to diminish, with similar prevalence observed between the two backhand techniques. This pattern is consistent with the hypothesis that using only the dominant arm in the 1HB may impose greater mechanical load on the elbow joint, particularly among players with less advanced technical skills.

Low back pain

Conversely, the results for LBP showed an opposite tendency. In intermediate and higher competitive levels (particularly TPRA and fourth category), the prevalence of low back pain was higher among players using the 1HB, with a statistically significant difference observed in one category (p = 0.0004). This finding indicates that, at least within that subgroup, players using a 1HB had a higher risk of LBP compared with those using a 2HB. In lower competitive levels and among professional players (ATP/WTA), no statistically significant differences were observed, likely due to the smaller number of participants in these subgroups.

## Discussion

The main finding of this study is that the backhand technique (1HB vs. 2HB) was not independently associated with either DEP or LBP after adjustment for age, sex, weekly training volume, and competitive level. Although crude analyses showed a higher prevalence of elbow pain among players using a 1HB, this difference disappeared in multivariable models, indicating the presence of a substantial confounding effect, particularly related to age and sex.

Because symptoms were self-reported, the term “tennis elbow” was avoided in the primary analyses, and the outcome was defined as DEP. Participants reporting elbow pain were also asked to specify whether symptoms were localized to the lateral (epicondylar) or medial (epitrochlear) region, and location-specific analyses were therefore conducted as secondary exploratory analyses. The decision to report elbow symptoms primarily as overall elbow pain is consistent with several epidemiological and questionnaire-based studies in tennis players, in which investigations frequently begin with the presence of elbow pain rather than a strict clinical diagnosis of tennis elbow or golfer’s elbow [7,33]. Moreover, population-based studies have shown that both lateral and medial epicondylopathy may contribute to elbow symptoms in physically active individuals [34].

Regarding DEP, age emerged as an independent predictor, with a progressive increase in prevalence observed with advancing age. This finding is consistent with the degenerative and cumulative nature of tendinopathy, which reflects chronic exposure to mechanical loading and a reduced adaptive capacity of tendon tissue.

An earlier study [35] reported a lifetime incidence of elbow pain of approximately 50% among adult tennis players older than 30 years. In another cross-sectional survey involving 529 adult recreational players from the United States Tennis Association (USTA) league, with a mean age of 46.9 years, the incidence of injuries was estimated at 3.0 injuries per 1,000 hours of play, with an overall prevalence of 53 injuries per 100 players [36]. In that study, the elbow represented the most common injury site (20% of all injuries), followed by the shoulder (15%). Consistent with these findings, the results of the present study suggest that age plays an important role in the predisposition to DEP in tennis, likely reflecting both lower technical proficiency at some playing levels and the cumulative effects of long-term mechanical loading.

Male sex was also independently associated with DEP. This observation may be explained by differences in stroke velocity, applied force, and cumulative playing volume rather than by isolated technical factors. Importantly, although the 1HB was significantly more common among male players, backhand type did not remain independently associated with elbow symptoms, suggesting that stroke technique alone does not represent a primary determinant of pain.

Regarding LBP, although biomechanical studies have demonstrated differences in trunk rotational demands between the 1HB and 2HB [21-24], these differences did not translate into a different clinical prevalence of symptoms in our sample. This finding suggests that kinematic variations observed in laboratory settings do not necessarily correspond to clinical outcomes, likely because they are mediated by factors such as muscular conditioning, neuromotor control, load management, and recovery capacity. Once again, these findings support a multifactorial model of injury risk in which technique represents only one of several contributing factors [7].

The analysis of the sample also revealed a progressive reduction in the use of the 1HB as the competitive level increased. This finding is consistent with the literature documenting the predominance of the 2HB in modern professional tennis and the technical evolution of the game toward greater stability and power during high-intensity rallies [20,24,37]. However, this transition was not associated with independent differences in the prevalence of the investigated conditions. This observation suggests that competitive level and playing experience may modulate the biomechanical impact of stroke execution more strongly than the technique itself, likely through improved physical adaptation and greater movement efficiency. Although some subgroup differences were observed, these findings should be interpreted with caution due to the exploratory nature of the analyses and the absence of adjustment for multiple comparisons.

Overall, the findings support the hypothesis that the risk of DEP and LBP in tennis is determined by a complex interaction between biological factors, cumulative load exposure, and individual characteristics rather than by the isolated choice of the backhand technique. From a clinical perspective, these results suggest that preventive strategies should primarily focus on load management, muscular conditioning, and global optimization of stroke mechanics rather than on isolated modifications of the backhand technique. These findings should be interpreted with caution due to the exploratory nature of the subgroup analyses.

Limitations

Given the cross-sectional design, the findings represent a snapshot in time and do not allow causal inferences to be drawn. This study has several limitations. Symptoms were self-reported without clinical confirmation, and the absence of standardized thresholds for pain intensity or duration may limit comparability with other studies. The non-random recruitment strategy may have introduced selection bias, and no a priori sample size calculation was performed. Survival bias may also be present, as players with severe symptoms may have modified their technique or discontinued tennis before participation. In addition, the subgroups defined by competitive level were not numerically uniform, with a relatively limited representation of professional players. Furthermore, the classification of the backhand technique was based on self-report rather than objective biomechanical assessment. Finally, relevant factors such as equipment characteristics (e.g., racket type, string tension), playing surface, and cumulative load were not assessed and may have contributed to residual confounding.

## Conclusions

In this study, the backhand technique was not independently associated with the prevalence of DEP or LBP after adjustment for age, sex, training volume, and competitive level. Although players using the 1HB showed a higher crude prevalence of elbow symptoms, this association was largely explained by confounding factors, particularly age and sex. These findings support a multifactorial model of injury risk in tennis, in which factors such as age, sex, cumulative load, and player characteristics appear to play a greater role than the isolated choice of the backhand technique.

## Appendices

Survey questionnaire administered to tennis players

This appendix presents the questionnaire used to collect demographic data, training characteristics, and musculoskeletal symptoms among tennis players participating in the study.

Section 1: Demographic Information

1. Age (years): ______

2. Sex: □ Male   □ Female

Section 2: Tennis Practice Characteristics

3. How many years have you been playing tennis?

   □ <1 year   □ 1-5 years   □ 6-10 years   □ >10 years

4. What is your current playing level?

   □ Beginner/Recreational player

   □ Amateur competitive player

   □ Third category

   □ Second category

   □ Professional (ATP/WTA circuit)

5. How many hours per week do you usually play or train? ______ hours/week

6. Which type of backhand do you primarily use?

   □ One‑handed backhand

   □ Two‑handed backhand

Section 3: Musculoskeletal Symptoms

7. During the past 12 months, have you experienced pain in the dominant elbow associated with playing tennis?

   □ Yes   □ No

8. If yes, where was the elbow pain located?

   □ Lateral (epicondylar region)

   □ Medial (epitrochlear region)

9. During the past 12 months, have you experienced low back pain associated with playing tennis?

   □ Yes   □ No

Note: Elbow symptoms were defined as dominant‑arm elbow pain (DEP). Participants reporting elbow pain were asked to specify the anatomical location (lateral epicondylar or medial epitrochlear region).
